# On Habits of Intoxication as Causing a Type of Disease

**Published:** 1860-04-01

**Authors:** 


### THE JOURNAL OF PSYCHOLOGICAL MEDICINE AND MENTAL PATHOLOGY.

APRIL 1, 1860.

### Art. I.—ON HABITS OF INTOXICATION AS CAUSING A TYPE OF DISEASE.

"Doctor, I will give you a wrinkle," said a friend to us not long ago, as we were gossiping concerning wine, and an attendant was directed to descend into the cellar, and bring from a particular bin a bottle of champagne. It may be as well, perhaps, to remark, lest the uninitiated should stumble over the term, that a *wrinkle*, in the refined slang of the day, signifies a little bit of practical wisdom. The phrase is highly metaphorical. Worldly wisdom increases with years; so also do the furrows which indent the forehead. Therefore an increase of wrinkles on the brow may be regarded as an index of increasing sapience, and *a* wrinkle may legitimately stand as a figurative synonym for an item of practical wisdom. This by the way. The champagne was in due time placed upon the table, and the sparkling fluid had a most agreeable taste and refreshing effect, for the evening was hot and stifling. "Well, how do you like the wine?" inquired our friend. "A pleasant drink for a scorching day," we replied. "Read that," he said, putting into our hands the cork which had just been extracted from the bottle, and pointing to the inner extremity—that which had been in proximity to the wine. There we saw and read, not a little to our astonishment, the formidable word MORT, printed in clear bold letters. "That," said our friend, "is a trade-mark, and when you see it affixed to the cork of a champagne bottle, you may rest assured that no grapes ever contributed towards the formation of the wine."

This was the *wrinkle;* but the singularity of the trade-mark awoke other thoughts than those immediately connected with the utility of knowing it. Should there have been a full stop after the different letters—each having a specific signification, the formation of the word being merely accidental? Or was the trade-mark such as we read it, *Mort—Death*—and if so, was its adoption a Mephistophelian satire of the wine-merchant on the wine vended? Truly, a reversed cork so stamped and placed upon the plates of the guests at a feast might well serve the purpose of the puppet-mummy introduced during the course of an ancient Egyptian banquet; or might convey as homely but as forcible a lesson as that taught in Holbein's drawing of the toper in the Dance of Death, in which Death is represented as officiously pouring the inebriating drink into the mouth of one of the carousers. Or may we regard this trade-mark as a foreshadowing of that time, which Dr. Magnus Huss tells us, in his work on *chronic alcoholism*, he doubts not will sooner or later arrive, when alcohol will no longer be known as *aqua vitæ* but *aqua mortis.*

And, indeed, as we become more and more familiar with the remote effects of alcohol upon the system, in whatever form the potent spirit be consumed, we cannot resist the conclusion that, as too commonly used, it would be more correctly termed *aqua mortis* than *aqua vitæ.* It will be well briefly to recal a few of the reasons which justify this inference, as in so doing we shall best pave the way to the subject-matter of this article.

Mr. Neison, in his researches on the "Rate of Mortality among Persons of Intemperate Habits," shows that in the instances he investigated, at the term of life 21—30 the mortality was upwards of *five times* that of the general community; and that in the succeeding twenty years of life it was about *four times* as great, the difference becoming less and less as age advanced. "If there be anything, therefore," he adds, "in the usages of society calculated to destroy life, the most powerful is certainly the inordinate use of strong drink."*

Again, of the immediate causes of death among the intemperate, Head diseases (Nervous system) ranked most prominent, while diseases of the Digestive organs and dropsy, and diseases of the Respiratory organs were about on a par, although showing a comparative difference of very considerable importance. Now the deaths from Head diseases among the population of England and Wales, aged twenty and upwards, constitute only 9·710 per cent. of the deaths from all causes at those ages; but Mr. Neison tells us that "among the intemperate classes they constitute 27·100 per cent., being nearly three times as great. With other diseases," continues this gentleman, "similar differences will be found. In the general community, the deaths from diseases of the respiratory organs, at the same period of life, amount to 33·150 per cent. of the

* *Vital Statistics*, 2nd edit. p. 205.

deaths from all causes; while among the intemperate group they are only 22·980 per cent. of all the deaths."* Of the Head diseases among the intemperate, upwards of 50 per cent. were recorded as "delirium tremens."

Further, while the mortality from the two groups of Head diseases and of the Digestive organs forms 15·950 per cent. of the deaths from all causes, at corresponding ages, the mortality from the same groups of diseases among the intemperate forms 50·40 per cent. of all the deaths that take place, or more than *three times the general average.* "These may, therefore," writes Mr. Neison, "be regarded as the distinctive type of the causes of death among intemperate persons; and the predominance of deaths assigned to such causes in any particular collection of facts, may fairly, in the absence of other and more direct evidence, add to the inference of irregularity of habits having prevailed to an unusual extent."†

If, then, the inordinate use of alcoholic beverages exercises so lethiferous an influence, it must be conceded on this ground alone, that the subject is one of sufficient gravity to claim the most careful attention of the practical physician. In our systems of medicine it is, however, customary chiefly to direct notice to the more immediate results of excessive indulgence, and these alone find a place in our nosological arrangements and mortality records. Not that the more remote effects of intemperance have been, or are overlooked or forgotten. Far from it; for the subject is one having such important bearings upon the moral, intellectual, and physical deterioration of individuals and of nations, and even upon the extinction of families and races, that much research has been and still is being devoted to it. And yet with all this the more remote morbific results of intemperance have not been so fully appreciated as to receive a legitimate position in systematic medicine, and until this requirement be fulfilled several evils must be perpetuated. First, the records of mortality and of sickness being thus far imperfect, from them no satisfactory measure can be obtained of the lethal or deteriorating influence of intemperance on the whole population. The nosological system adopted by the Registrar-General marks in two instances only—*intemperance* and *delirium tremens*—death as a result of excessive indulgence in strong drink. It is manifest that these examples can embrace but a small amount of the mortality arising directly from intoxication. The nosology of the Registrar-General is that, however, which principally governs the systematic teaching of physic in our schools of medicine. Hence a second evil, to wit, that the attention being fixed mainly upon the immediate ills arising from intemperance, we are apt to

* *Op. cit.*, p. 221. † *Op. cit.*, p. 222.

under-estimate the more remote and even more important, because less apparent, more insidious, and, in the end, more disastrous evils. Nor is this all; for the morbific effects which may be developed by the free usage of alcoholic drinks, short of intemperance, and which we think are still very imperfectly understood, are liable to be altogether overlooked in practical medicine.

The time has now come, however, when we think, that with no small advantage to medicine, and the community at large, the morbific effects of alcohol may rightly receive a more prominent position in the systematic teaching of the medical art than it has yet received. In 1852, Dr. Magnus Huss, of Stockholm, showed that the remote, as well as the immediate effects of intemperance upon the nervous system had a specific character, and that they could be clearly distinguished from other affections of the brain and spinal cord. The more remote effects of intemperance Dr. Magnus Huss designates by the term *chronic alcoholism*, by which he wished to be understood, "the collective symptoms of a disordered condition of the mental, motor, and sensory functions of the nervous system, these symptoms assuming a chronic form, and without their being immediately connected with any of those modifications of the central or peripheric portions of the nervous system which may be detected during life, or discovered after death by ocular inspection, such symptoms, however, affecting individuals who have persisted for a considerable length of time in the abuse of alcoholic liquors."

Adopting, then, the terminology of Dr. Magnus Huss, the poisonous effects of alcohol, in whatever form taken, would be designated by the general phrase *alcoholism*, these morbid results being further described as occurring either in an *acute* or *chronic* form. Or else, using the ordinary term *intoxication*, we should speak of acute and chronic alcoholic intoxication, including under the former phrase *drunkenness*, ordinarily so termed, and *delirium with tremor;* under the latter, the collective symptoms described by Dr. Magnus Huss.

Alcoholism is, perhaps, not a very euphonious term, but yet, for scientific usage, it is much preferable to intoxication. The latter is in common use to signify drunkenness, and its adoption as a technical phrase having a very different and much wider meaning than drunkenness, in the usual acceptation of that word, would most probably lead to confusion. It is well to avoid, if practicable, in scientific language, a word which has a technical and popular meaning differing the one from the other. Hence we prefer the term alcoholism.

A modification in the use of the term *chronic* alcoholism would, we think, also be advisable. The expression, as defined by Dr. Magnus Huss, and generally adopted by those who have written upon the subject, includes a wide range of symptoms, frequently differing greatly in intensity and duration, and to which the term *chronic* is not always very applicable. Dr. Leon Thomeuf, in a recent clinical essay (an inaugural thesis) on alcoholism,* describes three phases of the disorder, using the phrase alcoholic intoxication :—

1. *Acute alcoholic intoxication*, in which the effect is always immediately linked to the action of the cause, and this being exhausted, the disturbance to which it has given rise quickly vanishes. This, the more immediate or primitive phase of intoxication, includes drunkenness, oinomania, and delirium with tremor, and it is characterized by expansive sentiments, agitation, and fury.

2. *Subacute alcoholic intoxication*, which supervenes subsequently to the immediate action of the cause, and which corresponds sometimes to the alcoholic insanity of M. Marcel, at others to a peculiar form of melancholy, which is accompanied by certain disordered states of motility. This phase is marked by depressive sentiments.

3. *Chronic alcoholic intoxication*, which determines certain structural or functional changes in the central nervous system, giving rise to the ordinary symptoms of different forms of insanity.

Dr. Thomeuf's arrangement was derived from observation of cases at Charenton, of which asylum he was an *interne*, but we think that much advantage would be obtained from adopting this threefold division, and extending it to the whole phenomena of alcoholism, as observed as well in private and general practice, as in the special practice of a lunatic asylum.

Dr. B. A. Morel, in his recent important treatise on mental disorders, classes chronic alcoholism among the mental perversions produced by the use of divers inebriating matters. He writes—

"The continued and progressive ingestion of certain intoxicating substances, as, for example, alcohol, constitutes a malady which latterly has been designated *chronic alcoholism*. When we employ this term, we wish to design a pathological state, as well physical as moral, in an individual who, enjoying primitively his reason, suffers himself to slide progressively into habits which, becoming inveterate, present themselves in the form of irresistible tendencies, and determine in the organism lesions of a special nature. This does not, however, exclude the existence of other pathological, pre-existing conditions which influence the disposition that an individual may show to indulge to

* *Annales Medico-Psychologiques*, 1859, p. 565.

excess in alcoholic drinks. Thus, it is not uncommon to see, at the commencement of mental disorders, and particularly of general paralysis, persons who have always been cited for their sobriety, suddenly manifest great depravity of the instincts. Alcoholism, in cases of this kind, is a result of pre-existing disease; and, if this mode of considering the subject is of a character to throw light upon diagnosis and prognosis, it also has an important bearing in regard to the legal medicine of the insane."*

The question of the influence of alcoholism as a cause of insanity has been so recently raised, in the evidence given before the Parliamentary Committees which sat during the last and preceding sessions of Parliament, to inquire into the care and treatment of lunatics, that it may be useful to glance for a moment at the statistics in reference to this subject which have been noted by Dr. Thomeuf.

It would appear that 350 lunatics were admitted into Charenton in the two years 1857-58. Of these cases, 102 were caused principally, if not solely, by the use of alcoholic drinks, that is, 29·1 per cent. of the whole were cases of alcoholism. The different characters presented by these cases were carefully recorded and are of great interest. They were as follows:—

| | | | |
|---|---|---|---|
| Delirium tremens . | 15 per cent. | Melancholia . . . | |
| Drunken mania . | 6 „ | Anomalous psychical | 24 per cent. |
| Congestive mania . | 1 „ | symptoms, after | |
| General paralysis . | 34 „ | delirium tremens | 4 „ |
| Epileptiform convulsions . . . . | 2 „ | *Folie circularie* . . | 3 „ |
| | | *Stupidité* . . . . | 2 „ |

It will be understood from this list how it happens that, if the immediate cause of death in cases of alcoholism be the fact entered in our registers of mortality, which is the rule, the lethal effects of the disease will lie in a great measure hid among the records of deaths from idiopathic affections.

A requisite step to the admission of alcoholism in its fullest acceptation into our nosological arrangements, is a fuller appreciation than is yet generally possessed of the specific character of the subacute and chronic forms of the affection. Whatever tends to promote this end is of moment. We would, therefore, direct attention to a little work on the subject, which has recently been published by Dr. Marcet,† and which, although it professes chiefly to have a therapeutical object, is exceedingly well calculated to convey a clear notion of the nature and symptoms of chronic alco-

* *Traité des Maladies Mentales.* Par le Dr. B. A. Morel. Paris, 1860. p. 214.

† *On Chronic Alcoholic Intoxication, or Alcoholic Stimulants in connexion with the Nervous System:* with a Synoptical Table of Cases. By W. Marcet, M.D., F.R.S., Assistant-Physician to the Westminster Hospital, &c. Churchill, 1860.

holism (using that term with the signification attached to it by Dr. Magnus Huss), as this affection is most commonly met with.

"The symptoms of the disease," writes Dr. Marcet, "depend on a functional disturbance of the properties of the nervous system, which may last for weeks, months, or years, even after the habit of excessive drinking has been given up. On first applying to his medical adviser, the patient will probably not state the cause of his illness, and thus seriously mislead the physician in his estimation of the nature of the complaint. If we try to account for this difficulty of establishing the cause of the disease in cases of chronic alcoholism, it will be found that in some instances the patient is ashamed of his intemperance, and will not confess it. In others, he considers that the nature of his occupation is such as to require an excessive amount of drink; he is seldom or never drunk, in his opinion he takes no more than is absolutely required, and he is not aware of his suffering from alcoholic stimulants. Some will positively disbelieve that their illness can be owing to the abuse of alcoholic liquors, as they have been under a pledge to drink very little or none at all for some time previously; but it will be noticed, in the course of the examination, that before taking the pledge these individuals were thorough drunkards, and had been obliged to give up drinking on account of their health. Finally, in those instances where the mind has been affected through frequent fits of drunkenness and repeated attacks of delirium tremens, the patient may have great aversion from giving plain answers to the questions of the physician, and thus lead him to understand that he never indulged too freely in spirituous drinks.

"There is something peculiar in the look and gait of individuals in the habit of drinking to excess, or even of habitual tipplers, which will greatly assist in discovering the nature of the complaint, even before addressing the patient. His peculiar complexion, often sharp features, or, if he be fat, the injected cheeks and nose, and their violet appearance, the trembling of the limbs, often of the whole body, or a want of steadiness and co-ordination in the movements, not very unlike incipient chorea—all these are so many symptoms that the medical practitioner will not fail to observe. On conversing with such patients, their intellect will not often be found blunted, and the account they give of their sufferings is perhaps remarkably clear. It may be, however, that the patient has fallen into a state of melancholia, and fancies his doctor is attempting to do him some injury, in which case the sufferer will endeavour to turn aside the conversation, and adroitly avoid the subject. I have observed a well-marked instance of this kind in a boy of eighteen whose case I had great difficulty at first in making out; but a fortnight afterwards, his health having much improved, he gave me a clear account of his illness. Another patient, although he had given up the habit of drinking to excess, taking no more on an average than two pints of beer daily for the last six years, yet assured me that he was occasionally unconscious of what he was doing, and that his friends sometimes thought he was insane."—(pp. 6-9.)

The chief symptoms of chronic alcoholism are trembling of the feet and hands, gradually increasing weakness, and in the fully developed cases paralysis, startings of the tendons, and cramps. The sleep is much disturbed by frightful dreams, or it cannot be obtained either on account of formications or prickings, or neuralgic pains in the limbs, or extreme restlessness. In the more advanced stages of the disease, the night not unfrequently becomes a period of horrible suffering. The patient in vain seeks to place his limbs in a position which would afford relief to the uneasy sensations or burning pain which affects them, and if sleep or drowsiness steals upon him, it is presently driven away by convulsive startings, which seem to tear asunder the muscles. The neuralgic pains which at first haunt solely the night begin to affect the patient and increase upon him by day. Vertigo often happens, and at times the vision is clouded, or the patient feels as if he had been suddenly plunged into darkness. Hallucinations are of common occurrence. Dr. Marcet writes:—

"They mostly affect the organs of sight and of hearing. For instance, one of my patients, when walking in the street, had seen ropes dangling about his head; to another, objects appeared as if they were double; some perceived occasionally insects creeping about: the various visions often disappearing as soon as the attention was directed to them. These factitious perceptions of the sight appear sometimes so real that the individual moves aside to avoid an imaginary object standing in his way. A cabman (Case 47) I was treating for chronic alcoholism told me he frequently pulled up his horse suddenly, or drove to one side of the street, lest he should run over some obstacle he distinctly saw in front of his horse, and which he afterwards found not to exist in reality. In his case objects appeared to be multiplied to as many as ten times their real number, so that if a lamp-post, a man, or a cart, happened to be near him, he perceived ten lamp-posts, or ten men, or as many carts. He could not possibly make out which object was really to be avoided, and was obliged to give up driving on account of the risk of an accident. In most cases the patient is occasionally, or perhaps constantly, troubled with shadows or a black mist, or flying specks (*muscæ volitantes*) passing rapidly before his eyes, and causing a dimness of sight, especially when he is looking attentively at something; in the act of reading, for example, the book is suddenly darkened, and a state of almost complete blindness ensues, lasting a few minutes. I have met with one instance where the patient perceived spots of all kinds of colour.* During the long and sleepless nights, aberrations of the sight frequently happen. The wife of a patient I was treating for chronic alcoholism told me her husband often fancied, whilst lying awake, that he saw rats and cats, and various other de-

* Magnus Huss has observed cases of chronic alcoholism where objects appeared peculiarly coloured. He reports having met with two instances of hallucinations of the smell, and also with hallucinations of the taste, the patients believing they were drinking brandy instead of water.

scriptions of animals, on the bedclothes; he used to doze at intervals, and in the morning could not remember anything of the nightly visions. The aberrations of the sense of hearing are not so frequent, but I have met with patients who occasionally heard voices addressing them when nobody was present."—(pp. 11-14.)

The intellectual and moral faculties, particularly the latter, are invariably perverted to a greater or less extent, and too commonly the patient becomes completely debased and brutalized, heeding nothing but the cravings of his own appetites or the pricks of his own sufferings. The digestive functions are more or less interfered with, and the kidneys and liver are very liable to be morbidly affected, the former with granular degeneration. Frequently, also, the heart is enfeebled by fatty degeneration of its structure. Wasting sets in, and the patient in the end may sink from exhaustion, or an induced lesion of liver, kidneys, digestive organs or heart may cut his life short, or, which is the commonest result, he dies apoplectic, or maniacal, or demented, or paralysed from head to foot, intellect and feeling being first alike abolished.

Such are, briefly, the symptoms which commonly mark the progress of chronic alcoholism. They may not all be found to occur in each case, for every degree of intensity in the manifestation of the psychical or physical symptoms is found in different instances; but there never wants that grouping together of certain mental, motor, and sensory perversions which is characteristic of alcoholic poisoning. No better example could be given of a special form in which chronic alcoholism is sometimes observed, and of the significant grouping of particular symptoms already described, than the *alcoholic melancholia* remarked by Dr. Thomeuf, at Charenton. Fifteen instances came under the scrutiny of this writer, and the symptoms noted may be summed up as follows:—

*Psychical Symptoms.*—Hallucinations were the dominant symptom. Disordered sensations induced delirium, and were followed by perversion of the emotions and instincts. The hallucinations were of peculiar importance, because they may be regarded as pathognomonic of the affection. At the first glance they seemed to differ much: this patient beheld men who wished to assassinate him; that, while he sat at a *table-d'hôte*, heard voices mocking him; while another saw and felt vipers and toads which nipped him. But in almost every instance the hallucinations caused a painful moral impression, and often even profound terror.

Hallucinations of the hearing occurred twelve times in the fifteen cases; hallucinations of the sight eleven times; of the touch twice; and of the taste once.

Dr. Thomeuf never encountered in this affection an hallucination of a lively character, and the delirium always was of a melancholic and sombre cast. The delirious conceptions were marked by profound sadness—by ideas of persecution. Thus one patient accused himself of being a drunkard, and he sought to escape from this reproach by attempting to cast himself from the window. The tendency to suicide was, indeed, frequently observed. Another patient conceived that he had violated his daughter, and wished to deliver himself up to justice in order that he might be punished. A third heard voices which said to him that he was a wretch because he had had the small-pox; he believed himself to be rotten. Lastly, a fourth was convinced that he was no longer a man, but a woman, also a bitch.

The attention was always more or less affected. It was invariably necessary to elevate the voice in order to attract the patient's notice, or to repeat a question in order to procure an answer. The memory was generally enfeebled. The affections were either perverted or abolished, the patients hating those who were formerly most dear to them. Jealousy was occasionally a conspicuous passion, and the instincts themselves were not unfrequently perverted.

In the majority of the cases sleep was diminished, or even lost, during the whole progress of the malady. Frightful hallucinations or dreams, in several cases, prevented all rest at night. This is not difficult to be understood. Those who suffer from alcoholism are greatly depressed, and it is well known that a lowering of the vital powers gives to our ideas a tinge of sadness which is exaggerated by silence and particularly by darkness.

*Physical Symptoms.*—In twelve cases, Dr. Thomeuf observed marked quivering of the muscles of the face (this being generally more manifest on one side), an undulatory movement of the lip and trembling of the tongue. This is worthy of note, as usually the muscular tremblings of chronic alcoholism are most frequently observed in the limbs. In two cases these movements persisted for a year. Insensibility to pain is often observed in drunkenness, and the same phenomenon was noticed by Dr. Thomeuf as existing in several of his patients. In three he could plunge a needle into the limbs without exciting any sign of pain, and one of the patients bore the application of an enormous cautery to the arm without uttering a word. The insensibility was most apparent in the arms, beneath the elbows, and the legs, beneath the knees. The patients suffered also from dyspepsia, with acid eructations. The pulse in nearly every case was normal, varying from sixty to eighty-four beats, except during the exacerbations, when it became more rapid. Obscene ideas were occasionally noticed.

This affection invariably occurred among old drunkards, and it sometimes succeeded an attack of delirium tremens. The symptoms which usually ushered in the malady were characteristic of chronic alcoholism. The individual became pusillanimous, distrustful, and vindictive. There was trembling of the hands and arms, more manifest in the morning, and often the sole symptom. Then cephalalgia, vertigo, and dazzling of the eyes set in, with more or less disturbance of the digestive functions. If the affection was not checked here, melancholia, usually with paralytic symptoms, succeeded.

Almost the whole of Dr. Thomeuf's patients were discharged cured, or much relieved, after fifteen days' residence in the asylum. Generally, however, slight trembling of the muscles of the face and limbs remained. But old drunkards are commonly dipsomaniacs, and sooner or later graver symptoms are induced, and the patient's mental and moral faculties are utterly degraded or broken up, constituting a form of dementia known among our neighbours as *abrutissement des ivrognes*. Or, general paralysis supervenes, but not the general paralysis known as that of the insane. General paralysis, as a morbid entity, often occurs in persons who have been guilty of excess in spirituous liquors; but a distinction exists between the general paralysis which is a remote and ultimate result of alcoholism, and that which constitutes an idiopathic affection, having a well-marked course. Morel remarks, agreeing with Dr. Magnus Huss, that "It does not seem possible to us at the present day to confound chronic alcoholism with other idiopathic affections of the brain and spinal marrow. The progressive general paralysis of the insane, when it arrives at its last limits, is perhaps the sole affection of which the differential diagnosis offers some difficulty."* Calmeil, we presume, includes both forms of general paralysis under the head *chronic diffuse periencephalitis*, and that form which is an ultimate result of alcoholism will appertain to his melancholic type of the disease.† The etiological differences of the two varieties of the affection are, however, sufficiently important to render a knowledge of their differential characters useful. These Dr. Thomeuf sums up in the following manner:—

| *Alcoholic Melancholia complicated with Paralysis.* | *General Paralysis.* |
|---|---|
| From headache. | Generally as headache. |
| Active hallucinations affecting all the senses, disordered vision (illusions). | Enfeeblement of the understanding, rarely hallucinations. |

* *Traité des Dégénérescences*, p. 94.

† *Traité des Maladies Inflammatoires du Cerveau*, vol. i. p. 271, *et seq.*

| *Alcoholic Melancholia complicated with Paralysis.* | *General Paralysis.* |
|---|---|
| Delirious conceptions depending upon the hallucinations; ideas of persecution, tendency to suicide, evil instincts, consciousness of degradation. | Ideas of grandeur and contentment. |
| Embarrassed speech depending somewhat upon fear, upon startings of the muscles of the face, and especially upon tremulousness of the tongue. | Embarrassed speech depending upon feebleness of the conceptions and paralysis of the muscles of the face. |
| Feebleness little marked of the inferior members; equal on both sides. | Feebleness of the inferior members, more marked generally upon one side than the other. |
| Trembling of the hands and the arms more marked in the morning; formications, cramps, and startings of the tendons of the fore-arm. | Nothing appreciable in the superior limbs, sometimes default of co-ordination. |
| Pupils nearly always dilated. | Pupils often unequal, often contracted. |
| Anesthæsia of the extremities of the limbs, extending generally in the superior limbs to the elbow, and the inferior to the knee. | Sensibility normal or obtuse over the whole surface. |
| Sleep disturbed with dreams, sometimes sleeplessness. | Sleep generally normal. |
| Diminution of the appetite, acid eructations, vomiting of mucus in the morning. | Appetite augmented. |
| Diminution of the generative functions, frigidity. | Augmentation of the generative functions. |
| Readily cured or modified. | Progress of the disease ordinarily rapid, always fatal. |
| Occasional supervention of *delirium tremens.* | Tendency to congestions, and to epileptiform attacks."* |

The causes which predispose an individual to alcoholism are various. The quality of the spirituous fluid imbibed, the quantity, the time of imbibition, the age of the imbiber, the sex, the habits, and the profession, all exercise more or less influence in determining, or staving off, or modifying the ulterior results of intemperance or undue indulgence in strong drink. The whole of these points are clearly and pithily discussed by Dr. Marcet, to whose book we would refer our readers for information con-

* See Dr. Legrand du Saulle's Analysis of Dr. Thomeuf's Essay, *Annales Medico-Psychologiques*, 1859, p. 566, *et seq.*

cerning them. A few remarks, however, may not be amiss concerning one or two of the predisposing causes. And first of quality.

The recent commercial treaty with France, and the probable introduction of the lighter and commoner French wines into this country at a comparatively low cost, has given rise to a tolerably wide feeling of gratification in the kingdom. Not a little has been said in the Senate and elsewhere about the advantages to be derived from readiness of access to "a light, wholesome beverage," as compared with our own heady and too frequently adulterated beer. As a substitute for the port and sherry in common use, a cheap, light, good French wine would, no doubt, be a most welcome boon to many an individual, but what reason is there to believe, that if French wines should become a common drink among us, we should get the cheaper varieties any purer or better than the French themselves? Looking at the question solely from a health point of view, we obtain one or two facts from Dr. Thomeuf's essay, which are all the more worthy of being treasured up because they are of a recent date. He tells us that the subjects of alcoholism at Charenton had drunk chiefly brandy, *absinthe*, and *gros-vin*. Now Professor Bouchardat has stated, that the wine retailed in Paris is chiefly composed of a small quantity of wine, to which alcohol is added, with colouring matter. Hence Dr. Thomeuf concludes, that *brandy* is to be regarded as having been the ordinary drink of the patients he treated. Shall *we* escape from an inundation of "doctored" light wines?—wines, of which the "wholesomeness" would be most problematical.

Mr. Neison gives certain deductions concerning the influence of beer and spirits upon the mortality of the intemperate in this kingdom of great interest. He says, that the average duration of life after the commencement of intemperate habits is,

| | |
|---|---|
| Among beer-drinkers . . . | 21·7 years |
| „ spirit-drinkers . . . | 16·7 „ |
| And among those who drink spirits and beer indiscriminately . . | 16·1 „ |

Consequently the rate of mortality will be,

| | |
|---|---|
| Among beer-drinkers . . . | 4·597 per cent. yearly |
| „ spirit-drinkers . . . | 5·996 „ |
| „ mixed-drinkers . . . | 6·194 „ |

He adds, "Intemperate indulgence in the use of distilled liquors is hence more hurtful to health than the like use of fermented liquors, but the immoderate use of both combined is more injurious than the exclusive use of one kind only."*

* *Vital Statistics*, p. 218.

In illustration of the *quantity* which may occasionally induce alcoholism, Dr. Marcet cites a very curious case which came under his own observation:—

"G. B., aged 28, a stoker in the House of Parliament, admitted as out-patient at the Westminster Hospital, on February 24th, 1859. Has always been of sober habits, and was only drunk once in his life, when no more than twelve years of age. His daily allowance of beer has been one pint, and he has taken no spirits. Three years ago he became a teetotaller, because he found that even so little as one pint of beer daily did not agree with his health. He has suffered from the usual symptoms of chronic alcoholism for the last three years."—(p. 25.)

Mr. Neison has recorded several important deductions respecting the duration of life after the commencement of intemperate habits, among different classes of persons, which have an important bearing upon the question of the influence of *profession* upon alcoholism. It would appear from Mr. Neison's researches, that the average duration of life, in the intemperate, is—

| | | |
|---|---|---|
| Among | mechanics, working, and labouring men . | 18 years |
| ,, | traders, dealers, and merchants . . | 17 ,, |
| ,, | professional men and gentlemen . . . | 15 ,, |
| ,, | females . . . . . . . | 14 ,, |

Dr. Marcet remarks that, "women appear to be much less subject to suffer from the long-contracted abuse of alcoholic liquors than men" (p. 30). If the effect of the vicious habit be measured by the mortality, it will be seen, from Mr. Neison's data, that the reverse is probably the truth.

An attack of alcoholism may be determined in an intemperate person, either by an exceptional excess, or by the supervention of some disease, or the occurrence of an accident, diminishing the resistance of the system towards the poisonous effects of the spirit. Our own experience teaches us, that the influence of idiopathic affections in facilitating attacks of alcoholism should never be lost sight of in persons who live a free life, but who are not usually termed intemperate.

"There is in every class of society," writes Dr. Marcet, "a number of persons who, although they do not become intoxicated, suffer from chronic alcoholism, from drinking more spirits, wine, or beer than agrees with their health. Most of these persons lead a useful and active life, and apply for medical advice, being quite unaware of the cause of their illness. Many of the upper ranks of society are thus seized with symptoms of chronic alcoholism. The habit of indulging freely in wine at frequent dinner-parties, of drinking wine at lunch, of taking occasionally a glass of wine between meals, or of sipping every evening two or three glasses of sherry and water, or brandy and water; the usual good living at the officers' mess or at the clubs; the custom which exists for commercial travellers, not only of using freely stimulants at dinner, but also of drinking wine with their customers when transacting business, and finding, of course, an equal pleasure in these potations—all these various circumstances, and many others besides, are quite sufficient to bring on an attack of chronic alcoholism when an individual is predisposed to the disease. Drinking is not usually in these cases an indomitable habit, and accordingly, the patient will gladly give it up if he feel certain that by so doing his health can be improved."—(pp. 69-70.)

The *treatment* of chronic alcoholism has hitherto been chiefly hygienic and general—the hygiene being moral as well as physical. The control of the habit of drinking is only to be secured by obtaining the patient's complete confidence, or by secluding him, the latter resource being in the present state of the law, as a rule, admissible only when one or other of the ordinarily received forms of insanity has been developed by the pernicious vice. To this point we shall recur presently. Abstinence, entire abstinence from spirituous liquors, is the first necessity towards effecting a cure. It is requisite, also, that the depuratory organs should be placed in the completest order, by bringing to bear upon them the means, duly regulated according to the circumstances of the case, which the materiâ medicâ places at our disposal, preferring, however, as far as practicable, those which render the exhibition of drugs unnecessary. The diet, therefore, is of primary importance, and should be simple, nutritious, and readily digestible. The skin should receive peculiar attention and be acted upon by baths, warm or cold, sponging, friction, and regular exercise, according to the circumstances of the case. Acute pain may be combated by anodynes or opiates; but these will not be found to be of much utility by themselves, even if they be at all advisable, in extreme restlessness or delirium.

Prolonged warm baths, with or without cold effusion to the head, seem to be most serviceable when one or other of the events named occurs; and small doses of opium may be of service in procuring rest after the use of the bath. The whole armament of tonics have been brought to bear upon chronic alcoholism with more or less success.

Dr. Magnus Huss has recommended fusel-oil in the treatment of the affection. He gives the drug in the form of pills, and thinks that it diminishes the trembling, uneasiness, formications, and sense of debility. He recommends camphor also as a calmative.

Thus much for the general treatment of chronic alcoholism; but Dr. Marcet writes:—

"If chronic alcoholism be considered as depending on a peculiarly diseased condition of a certain part of the body, owing to the action of a poison, no remedy can be looked upon as decidedly efficacious unless it exerts its power not directly on the symptoms themselves, which are but the signs of the illness, but on the principle of the disorder. Bearing this in mind, I have endeavoured to discover a treatment which, by acting immediately on the nervous system, should remove its diseased condition—the result of the long-continued abuse of alcoholic stimulants, thereby acting as a means of arresting the symptoms of the illness. I am consequently not about to recommend one remedy for a certain symptom, and another remedy for another symptom, but shall endeavour to show that there exists a substance, possessed of powerful and definite medicinal properties, and having the remarkable property of restoring to health, or at all events of greatly relieving the disordered nervous system of persons suffering from chronic alcoholism; the medicinal agent in question acting efficaciously in cases where the principal symptom may be either sleeplessness, or hallucinations, or trembling, or any other; and this substance is OXIDE OF ZINC."—(pp. 76-77.)

Dr. Marcet enters at length into a consideration of the physiological and therapeutical action of this preparation, and the latter he conceives to be tonic, so far as the nervous system is concerned, sedative and antispasmodic. He states that he has used the metal with advantage in cases of epilepsy, chorea, mild hysteria, paralysis and lead-palsy, exhaustion from excessive mental work, and now also chronic alcoholism. At first he had looked upon the preparation as a specific agent in the treatment of epilepsy; but he now frankly states that he is "obliged to admit that it seldom, if ever, cures the disease, although its use is certainly often attended with beneficial effects."—(p. 95.)

Dr. Marcet describes the usual effects of the administration of the oxide of zinc in chronic alcoholism, within his experience, in the following terms:—

"First, the sleep is improved, the patient does not lie so long awake at night, and the nightmare becomes less frightful; then the hallucinations decrease, the patient is no longer troubled with black specks passing constantly before his eyes, or with the sight of imaginary objects, such as insects or other animals crawling about the room, and extraordinary noises are no longer heard; the attacks of trembling also diminish in frequency if not in intensity, and gradually pass off. This improvement is attended with an increase of appetite, as well as a marked diminution of the gastric symptoms; and when the patient can take food and digest it well, he may be looked upon as in a fair way towards recovery. Gradually, muscular power returns, and the mental depression, which frequently accompanies chronic alcoholism, disappears; the patient becomes cheerful and happy, and expresses with gratitude his joy at feeling quite well.* When the disorder is

* It is to be understood that during the period of recovery the symptoms are frequently not relieved in the above-mentioned order.

complicated with an organic disease, I have found it advisable to begin with oxide of zinc, in order to alleviate as much as possible the functional derangement of the nervous system, and then to adopt such a course of treatment as may be considered the most suitable to the occasion."—(pp. 104-105.)

Dr. Marcet supports his opinions on the therapeutical influence of the oxide of zinc in chronic alcoholism, by a detailed account of cases treated by him at the Westminster Hospital. He adds also a synoptical table of forty-eight cases that have been under his care, and of which twenty-five appear to have been cured and many of the others much relieved.

If the treatment of chronic alcoholism rested solely with the strictly medical relation between the physician and his patient, of which we have as yet alone spoken, we might look more hopefully than at present can be done, upon the effects of curative remedies in the graver forms of the disease. The frequent culmination of the affection, however, in one or other of the recognised forms of insanity often renders necessary the interference of the law in order effectually to deal with it by compulsory restraint. Now the law permits the seclusion of the different forms of mania or of melancholia, and so forth, resulting from intemperance, but it does not recognise the specific character of the mental aberration arising from that cause, but treats it in the same manner as idiopathic insanity. Neither does the law recognise intemperance as itself a frequent form of insanity, acquired or derived; nor does it recognise the chronic results of intemperance as a species of insanity *per se*. Hence the restraint which, by controlling the insatiable appetite for drink and the depraved desires of the patient, is essential in the treatment of the graver cases of alcoholism, can only be had recourse to during the delirious paroxysms or exacerbations of the affection, but cannot be applied to the affection itself. Thus a case of alcoholic homicidal or destructive mania is admitted into an asylum. The case rapidly improves, and presently the patient regains the appearance of one of sound mind. It is requisite thereupon that he should be at once discharged from control. If the case has resulted from an exceptional debauch, and the individual is not an habitual drinker, this is the right course to pursue; for it may be presumed that the morbid state of the mind has ceased entirely with the cessation of the symptoms of aberration. If, however, the patient be an habitual drinker, and more particularly if it appears that the habit is prompted by hereditary tendencies, and that the maniacal or melancholic state for which he was admitted into the asylum be but the culmination of a prolonged series of symptoms indicative of disordered emotional and instinctive powers, the case assumes a very different aspect. For the so-called attack of insanity, is under such circumstances, commonly but an exacerbation of chronic mental disorder. Hence if the relief of the exacerbation alone be looked upon as the cure of the disease, or as the justification for permitting the patient again to have self-control, sooner or later after discharge, he, driven by his insatiable and morbid appetite for drink, has once more recourse to it. What follows? The police records, or a walk through any asylum, will supply abundant information. The brutalized, insensate mass of flesh, sitting immovable and with idiotic aspect, in that corner of the corridor, had been thrice before admitted into the asylum for alcoholic mania, and in the intervals of his admissions he had ruined his family, driven his daughters into the streets, and his sons into crime. You may meet one or two of the former in the Haymarket, if you wish to confirm the story, any night; and one of the latter is to be found in a convict establishment, undergoing a long period of imprisonment. Again: we read, as we sip our matutinal coffee and munch our well-crisped toast, that A, B, or C, was discharged from this or that asylum, four or five weeks ago. He took a "little" drink the other night with a friend, on his way home stabbed a stranger to the heart, or, getting home, as the case may be, murdered his wife or one or two children, or himself—or *and* himself. If, however, he have avoided doing injury to himself, then the State steps in, a sufficiency of mischief having been fully and effectually accomplished, and shuts him carefully up for the remainder of his life. Rightly does the State recognise the murderous act as an insane one, and spare the scaffold; rightly does it prevent the possibility of such an act being committed again by the same individual; but wrongly, most wrongly, does it demur to make provision by which cases of insanity, acute or chronic, arising from the use of intoxicating liquids, might be kept a sufficient length of time under control, as would probably suffice to work a permanent effect upon the morbid appetite for strong drink, and upon the moral and intellectual debasement which invariably accompanies that appetite. The object sought, so far as the community is concerned, is to diminish as much as possible the probability of murder, suicide, or any other form of crime, or the ruin of families, being brought about in such a fashion; and this might be effected by specially providing for a greater latitude of detention in these cases than is usually accorded in cases of insanity. The Commissioners of Lunacy have suggested that they should be permitted to allow cases of insanity to leave asylums, when advisable, on trial, the original certificate of insanity being uncancelled. This provision would aid much in securing a more effectual treatment of alcoholic lunatics, and we hope that it will pass into law.

But the cases to which we have just referred—those which having been admitted into an asylum, are of necessity, from the existing state of the law, discharged before any permanent effect can be made upon the chronic mental perversion from which they suffer—constitute but a small portion of the difficulty which besets us in contending with insanity in its relations to intemperance. Certainly the said cases attract the chief attention both of the profession and the public, but there is an equally, and even if numbers be considered, more important class of cases in which medicine and morality is entirely helpless for lack of a power of legal interference. It is only, as we have already said, when the mental disorder from intemperance culminates in a commonly-recognised form of insanity, that the law interferes to save the individual from himself, and to protect his relatives and friends, or the community. During the whole of the nascent and maturing stages of the alienation, during the progressive degradation of the moral faculties of the individual, the medical man is compelled to look on, and witness the most heartrending ruin of a family, often in soul individually as well as in worldly possessions, by one whose intemperance is the manifestation of a true insane impulse, hereditary or acquired. The control of friends or relatives experience shows fails utterly, as a rule, to control the morbid propensity for drink and subtract the means for its indulgence—the initial steps towards either palliation or cure—and yet these cases cannot be consigned to an asylum. It has frequently been urged, and rightly so, that the law should permit the temporary seclusion of such examples of *habitual drunkenness* as those of which we have just spoken. And the public at once start back at the proposition. Daily familiar with the sight of drunkenness, they say, that if excessive drunkenness is to be made a criterion of madness, a door would be opened for the abuse of the law, and by means of which the most nefarious schemes could be carried on, as in the very worst period of the history of mad-houses. Even the hebdomadal, market-day toper would not be safe from incarceration through the officious kindness of anxious relatives or friends. The Law, also, properly chary of the liberty of the subject, may rightly object, that until medicine itself could show by something more than general descriptions the necessity for special legal intervention in reference to the insanity arising from intemperance, or to intemperance the result of insanity; until the records of our asylums, or our mortality tables bear upon their face indications of the proportion which the special forms of insanity arising from intemperance hold to idiopathic insanity; until the disease *alcoholism* in its fullest sense receives in our schools of medicine a measure of attention befitting the amount of social injury it is supposed to effect, the Law might reasonably doubt the necessity of interfering, and hesitate to do so. Interference may in the end be requisite, but it has not as yet been shown, to legal apprehensions, to be so.*

And this brings us back to another practical appreciation of the point from which we started at the commencement of this paper. Until the mental and physical phenomena of poisoning by alcohol receive an extension in the systematic teaching of medicine, and a position in our nosological arrangements in some degree pertinent to the amount of physical and moral mischief attributed to the potent liquid in ordinary life, we, the medical profession, shall probably not succeed in avoiding a habit of using phrases in reference to intoxication which to ourselves signify one thing, to the bystanders another; we shall not, therefore, succeed in convincing the public that intoxication or drunkenness the *mania*, and intoxication or drunkenness the bad habit, are two entirely different things—the one readily distinguishable, with care, from the other, and the one requiring the police magistrate, the other the doctor. Finally, we shall not convince the Law that *alcoholism* is a matter for special enactments, or rather for special provisions in the enactments for insanity generally.

In concluding this article we may remark, that Dr. Marcet's work, referred to several times in this article, constitutes the last addition to the literature of alcoholism, and it efficiently contributes, in so far as the more ordinary phenomena of the chronic affection is concerned, to a better knowledge of the subject. It is but just to him to add, that the work is brief, clear, very readable, and eminently practical—four properties of sufficient excellence to establish a prominent claim for notice of any book, apart from the consideration that this one has the additional excellence of recommending a new and tried plan of treatment, which, if it be confirmed by further experience, must prove a most valuable aid in dealing with a troublesome, and often impracticable disease.

---

### Art. II.—THE PLATONIC DIALOGUES.†

"Every one," writes Dr. Whewell, "that has any tinge of literature has heard of Socrates and Plato, who lived at Athens at the time of its greatest glory, when philosophy had its birth there." Yet it may be safely said, that until very lately few either

* Among the Flemings guardians are appointed over the persons and estates of prodigal individuals, as well as over lunatics.—*Southey's Common Place Book.*

